# Computational investigation of thermodynamic properties of gas phase vanadium nitride using Python

**DOI:** 10.1038/s41598-025-17514-z

**Published:** 2025-09-26

**Authors:** M. P. Yame, O. C. Akeremale, C. Iyen, S. J. Emem-Obong, T. V. Targema, B. J. Falaye

**Affiliations:** 1 Department of Physics, Faculty of Physical Sciences, Federal University of Lafia, P. M. B. 146, Lafia, Nigeria; 2 Department of Mathematics, Faculty of Physical Sciences, Federal University of Lafia, P. M. B. 146, Lafia, Nigeria; 3 Department of Pure and Applied Physics, Federal University Wukari, Wukari, Nigeria; 4 Department of Physics, Taraba State University, Jalingo, Nigeria; 5 Department of Physics, Anchor University, Lagos, Nigeria

**Keywords:** Vanadium nitride, Thermodynamic properties, Kratzer potential model, Vibrational partition function, Thermodynamics, Quantum physics

## Abstract

This study examines the thermodynamic characteristics of gas-phase diatomic vanadium nitride (VN(g)) utilizing the Kratzer potential model and Python as the computational tool. By solving the Schrödinger equation for the vibrational energy spectrum, we compute the vibrational partition function, which serves as the foundation for determining essential thermodynamic functions such as heat capacity ($C_p$), entropy ($S^\circ$), internal energy, and enthalpy. The calculated thermodynamic parameters are juxtaposed with experimental data from the NIST Chemistry WebBook to evaluate the precision of the Kratzer potential in simulating the thermal behavior of diatomic VN(g). Our results indicate that the Kratzer potential serves as a dependable approximation for the thermodynamic properties of molecular VN(g), especially in forecasting entropy and enthalpy over an extensive temperature range. The calculated heat capacity exhibits strong concordance with experimental data in the intermediate temperature range, though deviations appear at both low and high temperatures due to effects not captured by the model. Despite these limitations, the overall agreement validates the application of quantum molecular models, such as the Kratzer potential, in the thermodynamic analysis of transition-metal-containing diatomic molecules. This framework may be extended to similar gas-phase systems to support predictive modeling in molecular thermodynamics.

## Introduction

Vanadium nitride (VN) exists in various structural forms, including crystalline phases and gas-phase diatomic molecules. While solid-state VN has been extensively studied for its mechanical and electronic properties, this work focuses exclusively on the gas-phase diatomic VN molecule, which is relevant in high-temperature gas-phase reactions, combustion processes, and spectroscopic diagnostics.

Gas-phase diatomic VN is significant in high-temperature chemical environments, where its vibrational contributions to thermodynamic properties, such as entropy, heat capacity, internal energy, and enthalpy, are critical for understanding molecular-level energetics in vanadium–nitrogen systems. Unlike crystalline VN, which involves complex many-body interactions, the gas-phase VN molecule is well-approximated as a two-body quantum system.

The Kratzer potential, selected for its computational simplicity and ability to incorporate anharmonic corrections, enables effective modeling of VN(g)’s vibrational spectra^1–4^. Although the Morse potential is more widely used, it does not allow exact analytical solutions of the Schrödinger equation, often requiring numerical treatment of the eigenvalue problem. In contrast, the Kratzer potential yields closed-form expressions for vibrational energy levels, which can be directly utilized in numerical summation schemes for evaluating the partition function and related thermodynamic quantities. This combination of analytical energy expressions and numerical computation provides an efficient and accurate framework for thermodynamic modeling.

Compared to the Lennard-Jones potential, which is better suited for modeling weak intermolecular forces such as van der Waals interactions in non-bonded systems, the Kratzer potential is designed for bonded diatomic molecules and incorporates bond-specific parameters like equilibrium bond length and dissociation energy. While the Morse potential captures anharmonicity more realistically, its lack of analytical solvability limits its utility in purely theoretical derivations. The Kratzer potential thus offers a practical balance between physical realism and mathematical tractability for gas-phase vibrational modeling.

This model facilitates the derivation of closed-form energy expressions, forming the basis for evaluating the vibrational partition function and subsequent thermodynamic quantities. Other models, such as the Pöschl-Teller, Varshni, Deng–Fan potentials, etc., have also been successfully employed in similar studies^5–11^. Additionally, studies on analogous gas-phase diatomic molecules indicate that potential energy models, including the Kratzer potential, can accurately represent their thermophysical properties. The referenced models have not been applied to crystalline VN, and their relevance here is strictly limited to the molecular (gas-phase) VN system.

Experimental and computational studies provide critical benchmarks for validating theoretical approaches to gas-phase VN. Farber and Srivastava^12^ employed effusion–mass spectrometry over the temperature range 1900–2412 K to determine the formation enthalpy and dissociation energy of VN(g), reporting $\Delta H^\circ _{f,298} = 122.4 \pm 5$ kcal/mol and $D_0^\circ = 114.1$ kcal/mol. Recent *ab initio* configuration interaction calculations by Hendaoui *et al.*^13^ yielded accurate potential energy curves and spectroscopic constants for VN and related species, supporting the reliability of such methods for describing VN(g). Emission spectroscopy studies such as those by Fan *et al.*^14^ provide extensive data on VN(g)’s electronic–vibrational transitions, which are essential for constructing accurate quantum thermodynamic models.

In this study, the Schrödinger equation is solved using the Kratzer potential to derive vibrational energy levels for VN(g). These levels are used to construct the vibrational partition function, from which thermodynamic quantities are calculated over a wide temperature range. The computed results are compared with experimental data from the NIST Chemistry WebBook^15^ to assess the accuracy and limitations of the Kratzer model. The analysis is strictly limited to gas-phase VN, excluding crystalline, bulk, or nanostructured forms.

## Formulation of the problem

The thermodynamic properties of VN(g) can be explored within a quantum mechanical framework by solving the time-independent Schrödinger equation using a suitable interatomic potential. In this study, we adopt the Kratzer potential, a well-established model for describing molecular interactions in diatomic systems due to its ability to capture both the attractive and repulsive forces between atoms.

For a diatomic molecule such as VN(g), the system is governed by the time-independent Schrödinger equation^16–18^:

1 $$\begin{aligned} \hat{H} \Psi = E \Psi , \end{aligned}$$

where $\hat{H}$ is the Hamiltonian operator, *E* is the total energy of the system, and $\Psi$ is the corresponding wavefunction. The Hamiltonian for a vibrating diatomic molecule takes the form:

$$\begin{aligned} \hat{H} = -\frac{\hbar ^2}{2\mu } \nabla ^2 + V(r), \end{aligned}$$

where $\hbar$ is the reduced Planck constant, $\mu$ is the reduced mass of the vanadium and nitrogen atoms, and *V*(*r*) is the potential energy function that describes the interaction between the two nuclei.

The Kratzer potential, chosen for this work, is an anharmonic potential defined as^2–4^:

2 $$\begin{aligned} V(r) = D \left( \frac{r_0^2}{r^2} - \frac{2r_0}{r} \right) , \end{aligned}$$

where *D* is the dissociation energy; the energy required to break the VN(g) bond; and $r_0$ is the equilibrium bond length. This potential effectively captures the vibrational behavior of diatomic molecules and serves as a practical approximation for calculating thermodynamic properties in quantum systems, especially when considering molecules in the gas phase.

By solving the Schrödinger equation with the Kratzer potential using the formula method for bound state problems^19–21^, we obtain the vibrational energy spectrum of VN(g), which forms the foundation for computing its thermodynamic functions across a range of temperatures as:

3 $$\begin{aligned} E_{n,\ell } = -\frac{2\,\mu \,D^2\,r_0^2}{\hbar ^2 \left( \, n + \frac{1}{2} + \sqrt{\frac{2\,\mu \,D\,r_0^2}{\hbar ^2} + \left( \ell +\frac{1}{2}\right) ^2} \, \right) ^2}, \end{aligned}$$

where *n* is the principal quantum number and $\ell$ is the angular momentum quantum number. This equation provides the allowed discrete energy levels of the VN(g) molecule under the Kratzer potential, which is essential for computing thermodynamic functions. Since this solution have been obtained before using some other methods, we withhold the detailed calculations which lead to the above expression for the energy levels.

### Thermodynamics studies

The obtained energy levels serve as the foundation for evaluating thermodynamic functions. The vibrational partition function, denoted as $Q_{\text {vib}}(T)$, which is the sum over the Boltzmann-weighted vibrational states, is constructed as follows:

4 $$\begin{aligned} Q_{\text {vib}}(T) = \sum _{n} e^{- \beta (E_n - E_0)}, \end{aligned}$$

where $\beta = \frac{1}{k_B T}$ is the inverse thermal energy, $E_n$ represents the vibrational energy levels, computed from the Kratzer potential, $E_0$ is the ground-state vibrational energy. From this function, we can compute important thermodynamic quantities such as internal energy, entropy, specific heat capacity, and enthalpy. The partition function encapsulates the statistical behavior of VN(g) and provides a direct means to understand its temperature-dependent properties.

### Vibrational internal energy

The vibrational contribution to internal energy is computed as:

5 $$\begin{aligned} U_{\text {vib}}(T) = \frac{\sum _n E_n e^{- \beta (E_n - E_0)}}{\sum _n e^{- \beta (E_n - E_0)}}. \end{aligned}$$

This expression accounts for the thermal occupation of vibrational states. Since higher vibrational states become increasingly populated at elevated temperatures, the internal energy increases accordingly.

### Heat capacity at constant volume

The vibrational heat capacity, $C_V$, is derived from the variance of energy fluctuations:

6 $$\begin{aligned} C_{V, \text {vib}}(T) = \frac{1}{k_B T^2} \left( \langle E^2 \rangle - \langle E \rangle ^2 \right) , \end{aligned}$$

where $\langle E^2 \rangle = {\sum _n E_n^2 e^{- \beta (E_n - E_0)}}/{\sum _n e^{- \beta (E_n - E_0)}}$. This formulation ensures that $C_V$ correctly captures the temperature-dependent energy fluctuations within the vibrational system.

### Vibrational entropy

The vibrational entropy, denoted as $S_{\text {vib}}(T)$, quantifies the disorder associated with vibrational energy distributions:

7 $$\begin{aligned} S_{\text {vib}}(T) = R \ln Q_{\text {vib}} + \frac{U_{\text {vib}} N_A}{T}, \end{aligned}$$

where $R = N_A k_B$ is the universal gas constant. Entropy is an essential thermodynamic quantity as it governs the degree of molecular disorder and the system’s response to temperature variations.

### Translational contributions

Since VN exists as a gas-phase molecule under ideal conditions, its translational motion contributes significantly to its thermodynamic properties. The translational entropy is evaluated using the Sackur-Tetrode equation:

8 $$\begin{aligned} S_{\text {trans}}(T) = R \left[ \ln \left( \frac{(2\pi m k_B T)^{3/2} (k_B T)}{p_0 h^3} \right) + \frac{5}{2} \right] , \end{aligned}$$

where *m* is the molecular mass, and $p_0$ is the standard pressure. The translational internal energy follows from classical kinetic theory $U_{\text {trans}}(T) = \frac{3}{2} R T$, while the corresponding heat capacity remains constant $C_{V,\text {trans}}(T) = \frac{3}{2} R$. These expressions provide the translational contributions to the overall thermodynamic properties.

### Rotational contributions

For a diatomic molecule like VN(g), the rotational entropy follows the rigid-rotor approximation:

9 $$\begin{aligned} S_{\text {rot}}(T) = R \left[ \ln \left( \frac{T}{\Theta _{\text {rot}}} \right) + 1 \right] , \end{aligned}$$

where $\Theta _{\text {rot}}$ is the characteristic rotational temperature, given by $\Theta _{\text {rot}} = \frac{h^2}{8\pi ^2 I k_B}$. Here, *I* represents the moment of inertia of the VN(g) molecule. The rotational internal energy is $U_{\text {rot}}(T) = R T$ and the rotational heat capacity is $C_{V,\text {rot}}(T) = R$. These rotational contributions are added to the total thermodynamic properties.

### Total thermodynamic functions

The overall internal energy is obtained by summing over all contributions:

10 $$\begin{aligned} U_{\text {total}}(T) = U_{\text {trans}}(T) + U_{\text {rot}}(T) + U_{\text {vib}}(T). \end{aligned}$$

Similarly, the total heat capacity at constant volume is:

11 $$\begin{aligned} C_{V,\text {total}}(T) = C_{V,\text {trans}}(T) + C_{V,\text {rot}}(T) + C_{V,\text {vib}}(T). \end{aligned}$$

The total entropy follows as:

12 $$\begin{aligned} S_{\text {total}}(T) = S_{\text {trans}}(T) + S_{\text {rot}}(T) + S_{\text {vib}}(T). \end{aligned}$$

Finally, the enthalpy is computed as:

13 $$\begin{aligned} H_{\text {total}}(T) = U_{\text {total}}(T) + R T. \end{aligned}$$

**Table 1:** Calculated and experimental thermodynamic properties.

Temperature	C${}_p$ (This Study)	C${}_p$ (Experimental)	S${}^{\circ }$ (This Study)	S${}^{\circ }$ (Experimental)	H${}^{\circ }$ - H${}^\circ _{298.15}$ (This Study)	H${}^{\circ }$ - H${}^\circ _{298.15}$ (Experimental)
(K)	(J/mol$\cdot$K)	(J/mol$\cdot$K)	(J/mol$\cdot$K)	(J/mol$\cdot$K)	(kJ/mol)	(kJ/mol)
300	36.15	31.18	225.23	233.60	0.07	0.06
600	37.27	35.78	250.74	256.80	11.13	10.20
900	37.60	37.49	265.92	271.70	22.36	21.24
1200	37.80	38.14	276.76	282.60	33.67	32.59
1500	37.97	38.42	285.22	291.10	45.04	44.08
1800	38.13	38.68	292.15	298.20	56.45	55.64
2100	38.30	39.05	298.04	304.20	67.92	67.30
2400	38.47	39.56	303.17	309.40	79.43	79.08
2700	38.65	40.21	307.71	314.10	91.00	91.04
3000	38.84	40.98	311.79	318.40	102.62	103.20
3300	39.05	41.85	315.50	322.30	114.30	115.60
3600	39.27	42.80	318.91	326.00	126.05	128.30
3900	39.51	43.79	322.06	329.50	137.87	141.30
4200	39.76	44.79	325.00	332.70	149.76	154.60
4500	40.02	45.77	327.75	335.90	161.72	168.20
4800	40.29	46.71	330.34	338.90	173.77	182.10
5100	40.55	47.55	332.79	341.70	185.89	196.20
5400	40.81	48.29	335.12	344.50	198.10	210.60
5700	41.05	48.88	337.33	347.10	210.38	225.20
6000	41.28	49.29	339.44	349.60	222.73	239.90

### Computational approach

The thermodynamic properties of VN(g) are evaluated via a Python-based numerical method. We compute key thermodynamic quantities, including the vibrational partition function, entropy, internal energy, heat capacity, and enthalpy, over a wide temperature range. This computational framework integrates translational and rotational contributions to obtain a complete thermodynamic characterization of VN(g).

### Numerical implementation

The calculation process involves the following key steps:

1. First, we define physical constants and molecular parameters relevant to VN(g) as follows
  Planck’s constant: $$h = 6.62607015 \times 10^{-34}~\mathrm {J{\cdot }s}$$
  Reduced Planck’s constant: $\hbar = \frac{h}{2\pi } = 1.054571817 \times 10^{-34}~\mathrm {J{\cdot }s}$
  Boltzmann constant: $$k_B = 1.380649 \times 10^{-23}~\mathrm {J/K}$$
  Avogadro’s number: $$N_A = 6.02214076 \times 10^{23}~\mathrm {mol^{-1}}$$
  Ideal gas constant: $R = 8.31446261815324~\mathrm {J/(mol{\cdot }K)}$
  Atmospheric pressure: $$p_0 = 101325~\textrm{Pa}$$
  Conversion factor: $$1~\mathrm {cm^{-1}} = 1.98644586 \times 10^{-23}~\textrm{J}$$
  Atomic mass unit: $1~\textrm{u} = 1.66053906660 \times 10^{-27}~\textrm{kg}$
  Joule to eV: $1~\textrm{J} = 6.241509074 \times 10^{18}~\textrm{eV}$
  Reduced mass: $$\mu = 10.98~\textrm{u} = 1.8236 \times 10^{-26}~\textrm{kg}$$
  Dissociation energy (well depth): $D_e = 48000~\mathrm {cm^{-1}} = 9.5357 \times 10^{-19}~\textrm{J}$
  Equilibrium bond length: $r_e = 1.75 \times 10^{-10}~\textrm{m}$
  Molar mass of VN(g): $M = 0.064947~\mathrm {kg/mol}$
  These values are derived from standard references and computational data available in NIST Chemistry WebBook^15^.
2. Compute the vibrational energy levels using the Kratzer potential.
3. Evaluate the vibrational partition function to obtain thermodynamic properties.
4. Incorporate translational and rotational contributions.
5. Compute entropy, heat capacity, and enthalpy across various temperatures.
6. Compare results with experimental and theoretical values for validation.

The implementation is structured in modular functions, ensuring reproducibility and ease of extension. The numerical computations are performed over a wide temperature range ($T = 300$ K to 6000 K) using Python.

## Results and discussions

**Fig. 1:**
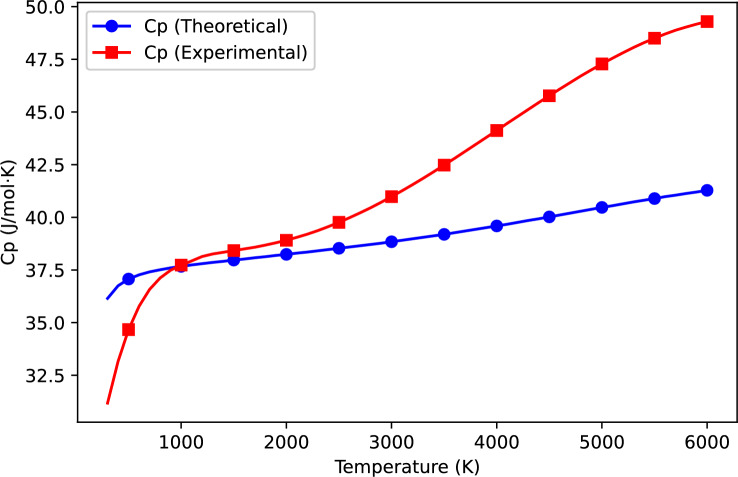
Temperature dependence of heat capacity ($C_p$) for VN(g). Theoretical predictions (blue) are compared with experimental data (red).

**Fig. 2:**
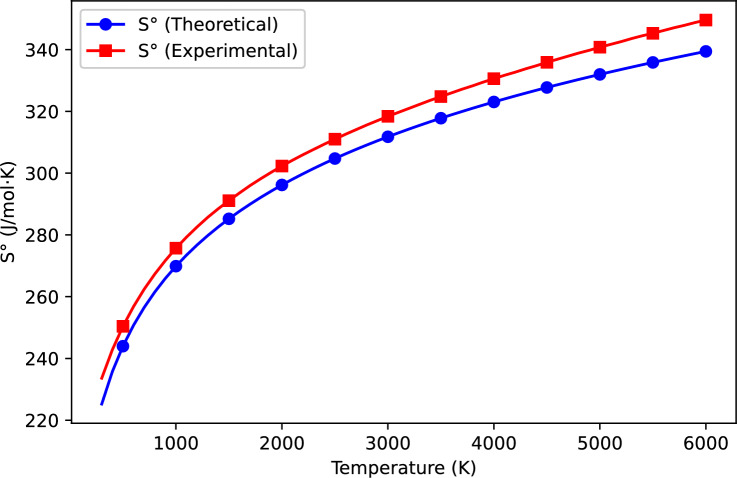
Comparison of computed and experimental entropy ($S^\circ$) as a function of temperature.

**Fig. 3:**
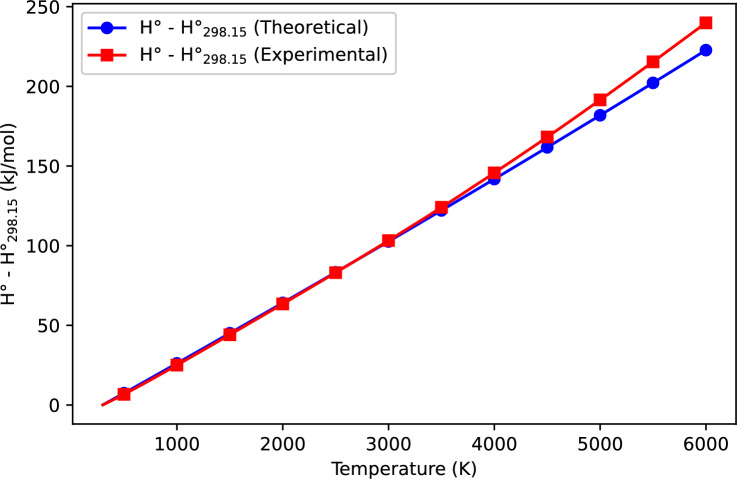
Temperature dependence of enthalpy change ($H^\circ - H^\circ _{298.15}$) for VN(g), comparing theoretical and experimental data^15^.

The thermodynamic properties of VN(g) were computed using the Kratzer potential model and compared with available experimental data^15^. Table 1 presents the computed and experimental values of heat capacity ($C_p$), entropy ($S^\circ$), and enthalpy change ($H^\circ - H^\circ _{298.15}$) over a wide temperature range. Figures 1, 2, and 3 further illustrate the variation of these properties with temperature, allowing for a direct comparison between theoretical predictions and experimental results.

### Heat capacity analysis

Heat capacity ($C_p$) is a central thermodynamic quantity that indicates the extent to which a substance absorbs heat as its temperature changes. Figure 1 compares the calculated vibrational heat capacity of VN(g) using the Kratzer potential with experimental data from the NIST Chemistry WebBook^15^.

The theoretical curve shows a monotonic increase in $C_p$ with temperature, consistent with general thermodynamic expectations. Agreement with experimental data is strongest in the intermediate temperature range (1000-3000 K), suggesting that the vibrational contribution is reasonably captured within this domain. However, notable deviations occur both below 1000 K and above 3000 K.

In the low-temperature region, the computed $C_p$ values systematically underestimate the experimental data. This discrepancy likely stems from the exclusion of rotational and translational degrees of freedom in the current model, which become especially important at lower temperatures. Moreover, the Kratzer potential (while incorporating anharmonicity) is primarily designed to capture vibrational dynamics and does not account for other excitations active in this regime.

At higher temperatures ($T > 3000\,\textrm{K}$), the model again underpredicts $C_p$. Three key effects may contribute to this:

- Anharmonic effects: The Kratzer potential, though effective for modeling vibrational interactions, assumes a near-harmonic behavior. However, real molecules experience anharmonicity, particularly at high temperatures, leading to an enhanced heat capacity^22,23^.
- Electronic contributions: At elevated temperatures, additional electronic energy levels become thermally accessible, increasing the overall energy absorption capacity of VN(g)^24^.
- Thermal expansion: Molecular bonds in VN(g) elongate with temperature, modifying the potential energy surface. The fixed equilibrium bond length assumed in the Kratzer potential neglects these effects, introducing discrepancies in predicted energy levels and thermodynamic quantities^25^.

In summary, while the Kratzer model provides a good description of vibrational contributions in the intermediate temperature range, its neglect of rotational, translational, and electronic degrees of freedom; as well as bond-length variations; limits its accuracy at temperature extremes. Although a quantitative assessment of these effects is beyond the scope of this study, their qualitative impact helps explain the observed deviations at both low and high temperatures.

### Entropy analysis

Entropy ($S^\circ$) represents the degree of disorder in a system and is a critical parameter in thermodynamics. Figure 2 illustrates the variation of entropy with temperature, comparing theoretical and experimental values. As expected, entropy increases monotonically with temperature due to the growing number of accessible microstates. The theoretical predictions slightly underestimate the experimental values, especially at high temperatures. This discrepancy may be attributed to:

- Higher-order vibrational states: The Kratzer potential, while incorporating anharmonicity, uses a finite number of vibrational energy levels. At elevated temperatures, real molecular systems may access higher vibrational states than included in the model, resulting in increased entropy.
- Additional degrees of freedom: In practical thermodynamic systems, total entropy includes translational, rotational, vibrational, and electronic contributions. At high temperatures, thermally accessible low-lying electronic states can contribute additional entropy, which is not captured in the vibrational-only model.

Nevertheless, the computed values remain within an acceptable range, supporting the applicability of the Kratzer potential for estimating the vibrational entropy of VN(g).

### Enthalpy change

Figure 3 presents the temperature-dependent enthalpy change ($H^\circ - H^\circ _{298.15}$), which represents the heat absorbed by the system relative to its enthalpy at 298.15 K. The enthalpy change increases almost linearly with temperature, which is consistent with thermodynamic expectations. Unlike $C_p$ and $S^\circ$, the computed and experimental enthalpy values show excellent agreement across all temperature ranges. This strong correlation suggests that the Kratzer potential effectively captures the energy absorption trends in VN(g).

### Overall model performance and limitations

Although the Kratzer potential offers a reliable framework for estimating the thermodynamic properties of VN(g), it is important to recognize certain limitations that may affect its accuracy:

- Neglect of anharmonicity: While the Kratzer model includes basic anharmonic features, it still approximates the molecular potential in a largely harmonic fashion. This simplification becomes less accurate at high temperatures, where higher vibrational states and stronger anharmonic effects are more pronounced.
- Absence of electronic contributions: The model focuses solely on vibrational interactions and does not consider electronic excitations, which can contribute significantly to the thermodynamic behavior of VN(g) at elevated temperatures.
- Experimental uncertainties: Some deviations between theoretical predictions and experimental observations may stem from measurement inaccuracies, variations in sample purity, or differences in experimental setups and conditions.

Despite these limitations, the Kratzer potential demonstrates commendable performance in modeling the thermodynamic behavior of gaseous VN. In particular, its predictions for enthalpy are more accurate than those for entropy and heat capacity. The reasons for this trend are further elaborated in the following section.

### Model performance: enthalpy versus other thermodynamic quantities

Among the thermodynamic properties examined, the Kratzer model exhibits the strongest agreement with experimental data for enthalpy. This can be attributed to the nature of how enthalpy is derived; primarily from the mean vibrational energy; which is relatively stable and less affected by minor inaccuracies in energy level spacing or the number of vibrational states included in the calculation.

In contrast, properties like entropy and heat capacity are more sensitive to the energy spacing. Their calculations involve logarithmic and derivative operations on the partition function, which tend to amplify small deviations, particularly at higher temperatures. At these elevated temperatures, contributions from higher vibrational levels and anharmonic effects become increasingly significant. However, the Kratzer potential, while incorporating basic anharmonicity, assumes a fixed bond length and only accounts for vibrational modes; excluding electronic excitations and thermal expansion; leading to larger deviations in computed values for $S^\circ$ and $C_p$.

The relatively stable behavior of enthalpy under such idealized modeling conditions explains the superior accuracy observed in our results. This robustness makes enthalpy a more reliable quantity when using simplified potentials like the Kratzer model to describe the thermodynamic properties of gaseous diatomic molecules such as VN(g).

## Conclusion

In this study, we have examined the thermodynamic properties of diatomic vanadium nitride in the gas phase using the Kratzer potential to model its vibrational interactions. By solving the Schrödinger equation, the vibrational energy levels were determined and subsequently used to compute the vibrational partition function. This formed the basis for calculating essential thermodynamic functions; heat capacity ($C_p$), entropy ($S^\circ$), and enthalpy change ($H^\circ - H^\circ _{298.15}$); over a wide temperature range. These theoretical results were compared with available experimental data^15^ to evaluate the performance of the Kratzer potential in modeling gaseous VN.

The findings indicate that the Kratzer potential offers a reasonable approximation for the vibrational behavior and thermodynamic characteristics of VN(g). The computed entropy and enthalpy values show strong agreement with experimental data across the examined temperature range, confirming the model’s effectiveness for gas-phase systems. While the calculated heat capacity matches well at lower temperatures, discrepancies arise at higher temperatures; primarily due to the model’s neglect of anharmonicity and electronic excitation effects, which are more pronounced in real molecular systems at elevated thermal energies.

Although the Kratzer potential captures the essential vibrational dynamics of VN gas, some inherent limitations should be noted. The model assumes a fixed bond length and does not incorporate anharmonic corrections, electronic contributions, or thermal expansion; all of which may introduce deviations in high-temperature predictions.

Nonetheless, this work reinforces the utility of the Kratzer potential as a computationally efficient tool for modeling the thermodynamic behavior of diatomic gases. It provides a reliable theoretical foundation for further explorations of similar transition metal nitrides in the gas phase.

In summary, this study contributes to a deeper understanding of the thermophysical properties of VN gas and demonstrates how simplified quantum models can be effectively employed in molecular thermodynamics. Future improvements incorporating anharmonicity, electronic structure, and rotational-vibrational coupling could enhance accuracy, particularly for high-temperature applications or more complex gas-phase systems.

## Supplementary Information

Supplementary Information.

## Data Availability

The computational data, including numerical results and Python scripts used for thermodynamic calculations, can be found as appendix to this manuscript. Experimental data used for validation were obtained from the NIST Chemistry WebBook and are publicly accessible at https://webbook.nist.gov/cgi/cbook.cgi?ID=C24646853&Units=SI&Mask=1 &Type=JANAFG&Table=on#JANAFG

## Code availability and reproducibility

The Python implementation allows for efficient computation of VN’s thermodynamic properties over a wide temperature range. The code can be adapted for other molecular systems by modifying the molecular parameters accordingly.
