# Supplementary material for: Computational investigation of thermodynamic properties of gas phase vanadium nitride using Python
Source: Sci Rep. 2025 Sep 26;15:33174. doi: 10.1038/s41598-025-17514-z (PMC12475087; doi:10.1038/s41598-025-17514-z)
Supplement: Supplementary file 1 — Supplementary Information. [file 41598_2025_17514_MOESM1_ESM.pdf]

Appendix A: Python Script for Thermodynamic Calculations

Below, we provide the Python implementation used for the computations. This script can be executed directly to obtain thermodynamic data for VN.

Listing 1: Python script for computing thermodynamic properties of VN using the Kratzer potential.

```python
import numpy as np

# ==============================
# Fundamental Constants (SI)
# ==============================
R    = 8.31446261815324         # J/(mol*K)
kB   = 1.380649e-23             # J/K
h    = 6.62607015e-34           # J*s
Na   = 6.02214076e23            # 1/mol
p0   = 101325                   # Pa (1 atm)
hbar = h / (2 * np.pi)

# Conversion factors
amu          = 1.66053906660e-27  # kg
cm1_to_J     = 1.98644586e-23     # 1 cm^-1 in Joules
J_TO_eV      = 1.0 / 1.602176634e-19  # Conversion factor: Joules -> eV

# ---------------------------
# Kratzer Energy Function
# ---------------------------
def kratzer_energy(n, l, mu, D, r0):
    """
    Computes the Kratzer energy level:
      E = - (2 * mu * D^2 * r0^2 / hbar^2) *
                [ n + 1/2 + sqrt( (2*mu*D*r0^2)/hbar^2 + (l+1/2)^2 ) ]^-2

    Parameters
    ----------
    n    : radial quantum number (integer)
    l    : angular quantum number (integer)
    mu   : reduced mass (kg)
    D    : well depth (J)
    r0   : equilibrium bond distance (m)

    Returns
    -------
    E    : energy (J)
    """
    term_inside = n + 0.5 + np.sqrt((2.0 * mu * D * r0**2)/(hbar**2) + (l + 0.5)
       **2)
    numerator   = 2.0 * mu * (D**2) * (r0**2)
    denom       = (hbar**2) * (term_inside**2)
    return - numerator / denom

# ---------------------------
# Molecular Data for VN Only
# ---------------------------
molecules_data = {
    "VN":  (10.98, 48000.0),  # (reduced mass in amu, well depth in cm^-1)
}

# Equilibrium bond distance (r0) in meters for VN
molecule_bond_lengths = {
    "VN":  1.75e-10,  # m
```

```
}

# Molar mass (kg/mol) for VN (used for rotational contributions)
molecule_molar_masses = {
    "VN":  0.064947,  # kg/mol
}

# ---------------------------
# Define the set of (n, l) states for Table 1
# ---------------------------
states = [
    (0, 0),
    (1, 0), (1, 1),
    (2, 0), (2, 1), (2, 2),
    (3, 0), (3, 1), (3, 2), (3, 3),
    (4, 0), (4, 1), (4, 2), (4, 3), (4, 4),
    (5, 0), (5, 1), (5, 2), (5, 3), (5, 4), (5, 5)
]

# ---------------------------
# Table 1: Compute E(n,l) (in eV) for VN using the Kratzer potential
# ---------------------------
energies_table = {}

for mol, (mu_amu, _, D_cm1) in molecules_data.items():
    mu_kg = mu_amu * amu
    r0    = molecule_bond_lengths[mol]
    D_J   = D_cm1 * cm1_to_J  # Convert well depth from cm^-1 to Joules
    energies = []
    for (n, l) in states:
        E_nl = kratzer_energy(n, l, mu_kg, D_J, r0)
        energies.append(E_nl)
    energies_table[mol] = energies

# Print Table 1 (energies in eV) for VN
print("Table 1: Kratzer Energy Eigenvalues E(n,l) (eV) for VN")
header = " n   | l   || " + "  |  ".join([f"E({mol})" for mol in molecules_data.
   keys()])
print(header)
print("-" * len(header))
for idx, (n, l) in enumerate(states):
    row_vals = []
    for mol in molecules_data.keys():
        energy_eV = energies_table[mol][idx] * J_TO_eV
        row_vals.append(f"{energy_eV:12.4e}")
    print(f"{n:2d} | {l:2d} || " + "  ".join(row_vals))

# ---------------------------
# Functions for Thermodynamic Properties Using Kratzer Vibrational Levels (VN)
# ---------------------------
def get_vibrational_levels_kratzer(mu, D, r0):
    """
    Compute the vibrational (l=0) energy levels for the Kratzer potential.
    The maximum vibrational quantum number is given by:
      n_max = floor( sqrt((2*mu*D*r0^2)/hbar^2 + 0.25) - 0.5 ).

    Parameters
    ----------
    mu : reduced mass (kg)
    D  : well depth (J)
    r0 : equilibrium bond distance (m)
```

```
    Returns
    -------
    levels : numpy array of vibrational energy levels (J) for n=0,1,...,n_max
    """
    alpha = np.sqrt((2.0 * mu * D * r0**2) / (hbar**2))
    n_max = int(np.floor(np.sqrt(alpha**2 + 0.25) - 0.5))
    levels = np.array([kratzer_energy(n, 0, mu, D, r0) for n in range(n_max + 1)])
    return levels

def Q_vib_kratzer(T, levels):
    beta = 1.0 / (kB * T)
    E_shift = levels - levels[0]
    return np.sum(np.exp(-beta * E_shift))

def U_vib_kratzer(T, levels):
    beta = 1.0 / (kB * T)
    E_shift = levels - levels[0]
    Q = np.sum(np.exp(-beta * E_shift))
    U = np.sum(E_shift * np.exp(-beta * E_shift)) / Q
    return U  # per molecule (J)

def E2_avg_kratzer(T, levels):
    beta = 1.0 / (kB * T)
    E_shift = levels - levels[0]
    Q = np.sum(np.exp(-beta * E_shift))
    return np.sum(E_shift**2 * np.exp(-beta * E_shift)) / Q

def Cv_vib_kratzer(T, levels):
    U = U_vib_kratzer(T, levels)
    E2 = E2_avg_kratzer(T, levels)
    Cv = (E2 - U**2) / (kB * T**2)
    return Cv * Na  # per mole (J/mol*K)

def S_vib_kratzer(T, levels):
    beta = 1.0 / (kB * T)
    Q = Q_vib_kratzer(T, levels)
    U = U_vib_kratzer(T, levels)
    S = R * np.log(Q) + (U * Na) / T
    return S

# Translational contributions (per mole)
def translational_entropy(T, molecule_mass, p=p0):
    term = (2*np.pi*molecule_mass*kB*T/h**2)**1.5
    return R * (np.log(term * (kB*T/p)) + 5/2)

def translational_internal_energy(T):
    return 1.5 * R * T

def translational_Cv(T):
    return 1.5 * R

# Rotational contributions (rigid-rotor, linear molecule)
def rotational_entropy(T, theta_rot):
    return R * (np.log(T/theta_rot) + 1)

def rotational_internal_energy(T):
    return R * T

def rotational_Cv(T):
    return R
```

```python
# Total Thermodynamic Properties (per mole)
def total_internal_energy(T, molecule_mass, theta_rot, vib_levels):
    U_trans = translational_internal_energy(T)
    U_rot   = rotational_internal_energy(T)
    U_vib   = U_vib_kratzer(T, vib_levels) * Na
    return U_trans + U_rot + U_vib

def total_Cv(T, molecule_mass, theta_rot, vib_levels):
    Cv_trans = translational_Cv(T)
    Cv_rot   = rotational_Cv(T)
    Cv_vib   = Cv_vib_kratzer(T, vib_levels)
    return Cv_trans + Cv_rot + Cv_vib

def total_entropy(T, molecule_mass, theta_rot, vib_levels, p=p0):
    S_trans = translational_entropy(T, molecule_mass, p)
    S_rot   = rotational_entropy(T, theta_rot)
    S_vib   = S_vib_kratzer(T, vib_levels)
    return S_trans + S_rot + S_vib

def total_enthalpy(T, molecule_mass, theta_rot, vib_levels):
    return total_internal_energy(T, molecule_mass, theta_rot, vib_levels) + R * T

# ---------------------------
# Temperature Grids for Thermodynamic Tables (VN)
# ---------------------------
T_vals1 = np.array([298, 300, 400, 500, 600, 700, 800, 900, 1000, 1100])
T_vals2 = np.arange(1100, 6001, 100)

# ---------------------------
# Compute and Print Thermodynamic Properties for VN using Kratzer Potential
# ---------------------------
T_ref = 298.15  # reference temperature in K

for mol in molecules_data.keys():
    print("\n" + "="*60)
    print(f"Thermodynamic Properties for {mol} (Kratzer Potential)")
    print("="*60)

    # Get molecule-specific parameters for VN:
    mu_amu, _, D_cm1 = molecules_data[mol]
    mu_kg = mu_amu * amu
    r0    = molecule_bond_lengths[mol]
    D_J   = D_cm1 * cm1_to_J

    # For rotational part:
    M_mol = molecule_molar_masses[mol]
    molecule_mass = M_mol / Na
    I = mu_kg * r0**2
    theta_rot = h**2 / (8 * np.pi**2 * I * kB)

    # Vibrational levels (l=0) for the Kratzer potential:
    vib_levels = get_vibrational_levels_kratzer(mu_kg, D_J, r0)

    # Reference enthalpy at T_ref:
    H_ref = total_enthalpy(T_ref, molecule_mass, theta_rot, vib_levels)

    # --- Table for T_vals1 ---
    print("\n-- Temperature Range: 298 K to 1100 K --")
    print("T (K)\tCp (J/mol*K)\tS (J/mol*K)\t?H (kJ/mol)")
    for T in T_vals1:
```

```
    Cp = total_Cv(T, molecule_mass, theta_rot, vib_levels) + R  # Cp = Cv + R
        for an ideal gas
    S  = total_entropy(T, molecule_mass, theta_rot, vib_levels)
    H  = total_enthalpy(T, molecule_mass, theta_rot, vib_levels)
    dH = (H - H_ref) / 1000.0
    print(f"{T:7.2f}\t{Cp:14.2f}\t{S:14.2f}\t{dH:14.2f}")

# --- Table for T_vals2 ---
print("\n-- Temperature Range: 1100 K to 6000 K --")
print("T (K)\tCp (J/mol*K)\tS (J/mol*K)\t?H (kJ/mol)")
for T in T_vals2:
    Cp = total_Cv(T, molecule_mass, theta_rot, vib_levels) + R
    S  = total_entropy(T, molecule_mass, theta_rot, vib_levels)
    H  = total_enthalpy(T, molecule_mass, theta_rot, vib_levels)
    dH = (H - H_ref) / 1000.0
    print(f"{T:7.2f}\t{Cp:14.2f}\t{S:14.2f}\t{dH:14.2f}")
```
